# *Williamsia muralis* Pulmonary Infection

**DOI:** 10.3201/eid1108.050439

**Published:** 2005-08

**Authors:** Maria del Mar Tomas, Rita Moure, Juan Antonio Saez Nieto, Salvador Fojon, Ana Fernandez, Maria Diaz, Rosa Villanueva, German Bou

**Affiliations:** * Complejo Hospitalario Universitario Juan Canalejo, La Coruña, Spain;; † Centro Nacional de Microbiologia, ISCIII, Majadahonda, Madrid, Spain

**Keywords:** Emerging, Pathogen, Williamsia muralis

**To the Editor:** Bacteria of the genus *Williamsia* are mycolic acid–containing actinomycetes of the suborder Corynebacterineae (1). This suborder also includes the genera *Gordonia*, *Mycobacterium*, *Nocardia*, *Corynebacterium*, *Rhodococcus*, *Dietzia*, *Skermania*, *Tsukamurella*, and *Turicella* (2,3). Within the genus *Williamsia*, only 2 species have been reported: *Williamsia muralis*, isolated from a daycare center (4), and *W*. *maris*, isolated from the Sea of Japan (5). One important aspect shared by both species is their apparent lack of pathogenicity, since they have been isolated only from environmental samples.

An 80-year-old woman, whose medical history included allergy to penicillin and high blood pressure, was admitted to the cardiothoracic intensive care unit at Juan Canalejo Hospital Complex in La Coruña, Spain, because of a loss of consciousness following an aortic valve replacement. Physical examination showed a systolic murmur and an echocardiogram showed aortic stenosis. Transaortic peak pressure was 100 mm Hg, and the aortic valvular area was 0.3 cm^2^. A biologic valve prosthesis (Mitroflow 21, Sorin Group Canada, Ltd., Burnaby, British Columbia, Canada) was inserted under the cardiopulmonary bypass.

Forty-eight hours later, the patient had paroxysmal atrial fibrillation and a temperature of 39°C, with severe hemodynamic and respiratory impairment. She was intubated and intravenous drugs were administered. Blood and urine cultures were requested. Central venous pressure lines were changed, and cultures were obtained. Empiric treatment with levofloxacin, amikacin, and teicoplanin was started for the patient. One of 2 blood cultures was positive for *Staphylococcus epidermidis*, as were cultures from femoral and jugular venous lines. Although considered a contaminant, we observed that *S. epidermidis* was susceptible to empiric antimicrobial drugs.

One week later, a chest radiograph showed bilateral alveolar infiltrates suggestive of pulmonary edema (Figure). To rule out infection, bronchoscopy and protected specimen brush were conducted. An unidentified gram-positive bacillus was cultured from the brush sample. Urine cultures were positive for *Candida kefyr*, but the patient showed no evidence of candidemia. An echocardiogram showed no evidence of infective endocarditis. Since the patient's condition did not improve, levofloxacin was replaced with imipenem, and treatment with fluconazole was initiated. However, the patient developed septic shock, adult respiratory distress syndrome, and oliguric acute renal failure, and died of multiple organ failure.

**Figure:**
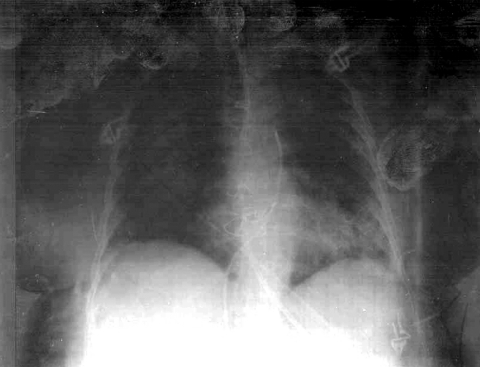
Chest radiograph of the patient showing bilateral alveolar infiltrates. Although pulmonary edema was the initial diagnosis, an infectious cause should be considered and, on the basis of sepsis, appropriate treatment initiated.

On direct examination, a Gram stain of the protected specimen brush sample showed numerous gram-positive bacilli. After incubation for 48 h in either an aerobic or capnophilic atmosphere, >1,000 CFU/mL were observed on Columbia agar plates containing 5% sheep blood (BD Stacker Plates, BBL, Franklin Lakes, NJ, USA). These colonies were round, slightly convex, white to pale yellow, and 1–3 mm in diameter. Microscopic examination showed short gram-positive bacilli. A few colonies of coagulase-negative staphylococci were also isolated from the clinical sample.

We attempted biochemical identification of the gram-positive bacilli, but discordant results were obtained. Test results of cultures after 24 and 48 h with a commercial assay (apiCoryne, bioMérieux, Marcy l'Etoile, France) identified the bacilli as a *Rhodococcus* spp. (probability >98.2%). However, negative results by Kinyoun modified acid-fast staining and by the CAMP test, 2 features characteristic of *Rhodococcus equi*, aroused suspicion regarding the unusual properties of this isolate.

Genomic DNA was isolated from the bacilli and analyzed by polymerase chain reaction (PCR)–mediated amplification of 16S ribosomal DNA, purification of PCR products, and direct sequencing, as previously reported (6). The 16S rRNA gene sequence (1,438 bp) of the isolate (500/04; GenBank accession no. AY986734) showed 99% similarity with *W. muralis* (4). Other noteworthy similarity matches of the isolate were with *W. maris* (96%), *Gordonia* sp. (95%), *Nocardia transvalensis* (95%), and *Rhodococcus* sp. (95%).

Antimicrobial drug susceptibility patterns were determined by using a commercial assay (Trek Diagnostic Systems Ltd., East Grinstead, UK). Since no interpretive criteria exist for *Williamsia* spp., those previously reported for *Nocardia* spp (7,8). were used for estimating breakpoints. Results showed the isolate was susceptible to amoxicillin-clavulanate, cefotaxime, imipenem, ciprofloxacin, tobramycin, gentamicin, and cotrimoxazole and resistant to ampicillin and erythromycin after incubation for 48 h in either air or a CO_2_ atmosphere (GasPak CO_2_ Pouch Capnophilic System, BD Biosciences, Sparks, MD, USA). Isolates of the genus *Williamsia* are currently recognized as environmental microorganisms (4,5). However, its potential as a pathogen in clinical infections has not been reported.

In summary, we report the isolation of >1,000 CFU/mL of *W*. *muralis* from a protected specimen brush sample of an 80-year-old woman. The number of colonies obtained, as well as features of the source of the clinical sample and the chest radiograph (clearly pathologic) at the time the isolate was obtained, strongly suggest that this microorganism was associated with lung infiltrates and poor prognosis, resulting in the death of the patient. The isolation of a few colonies of a coagulase-negative staphylococci may be considered irrelevant.

We also report the antimicrobial drug susceptibility pattern of *Williamsia* spp. Since no clinical findings for this genus have been reported, no clinical recommendations have been made regarding empiric treatment for infections with this microorganism. This is the first report of this bacterium as a potential human pathogen.

This work was partially supported by RESITRA (G03/75).
